# Transcriptomic complexity in young maize primary roots in response to low water potentials

**DOI:** 10.1186/1471-2164-15-741

**Published:** 2014-08-29

**Authors:** Nina Opitz, Anja Paschold, Caroline Marcon, Waqas Ahmed Malik, Christa Lanz, Hans-Peter Piepho, Frank Hochholdinger

**Affiliations:** Institute of Crop Science and Resource Conservation (INRES), Crop Functional Genomics, University of Bonn, 53113 Bonn, Germany; Institute for Crop Science, Bioinformatics Unit, University of Hohenheim, 70599 Stuttgart, Germany; Department of Molecular Biology, Max-Planck-Institute for Developmental Biology, 72076 Tübingen, Germany

**Keywords:** Drought, Low water potential, Maize, RNA-Seq, Root, Transcriptome, Water deficit

## Abstract

**Background:**

Widespread and more frequently occurring drought conditions are a consequence of global warming and increase the demand for tolerant crop varieties to feed the growing world population. A better understanding of the molecular mechanisms underlying the water deficit response of crops will enable targeted breeding strategies to develop robust cultivars.

**Results:**

In the present study, the transcriptional response of maize (*Zea mays* L.) primary roots to low water potentials was monitored by RNA sequencing (RNA-Seq) experiments. After 6 h and 24 h of mild (-0.2 MPa) and severe (-0.8 MPa) water deficit conditions, the primary root transcriptomes of seedlings grown under water deficit and control conditions were compared. The number of responsive genes was dependent on and increased with intensification of water deficit treatment. After short-term mild and severe water deficit 249 and 3,000 genes were differentially expressed, respectively. After a 24 h treatment the number of affected genes increased to 7,267 and 12,838 for mild and severe water deficit, respectively, including more than 80% of the short-term responsive genes. About half of the differentially expressed genes were up-regulated and maximal fold-changes increased with treatment intensity to more than 300-fold. A consensus set of 53 genes was differentially regulated independently of the nature of deficit treatment. Characterization revealed an overrepresentation of the Gene Ontology (GO) categories “oxidoreductase activity” and “heme binding” among regulated genes connecting the water deficit response to ROS metabolism.

**Conclusion:**

This study gives a comprehensive insight in water deficit responsive genes in young maize primary roots and provides a set of candidate genes that merit further genetic analyses in the future.

**Electronic supplementary material:**

The online version of this article (doi:10.1186/1471-2164-15-741) contains supplementary material, which is available to authorized users.

## Background

Population growth and global warming are major challenges for global food security. It is estimated that the demand for agricultural products will increase by ~50% until 2030 [1]. This requires historically unprecedented annual production growth rates [2]. Worldwide, about 70% of the food production is provided by cereals [3]. Maize (*Zea mays* L.) outcompeted all other cereals with an estimated global yield of 863 million tons in 2012/2013 [4]. While cereal production needs to be significantly increased, climate change adversely affects global maize production with an estimated loss of ~4% relative to what could have been achieved without the climate trends [5]. Poor soil moisture is widespread among arable land and as a consequence of global warming more areas are affected by drought conditions each year [6]. Since water availability is the most critical environmental factor for plant growth [7], drought can limit crop productivity more than any other abiotic stress. Furthermore, variations in water availability within fields can result in uneven crop stands that cause yield losses [8].

Under drought conditions, when water loss through transpiration is high, it is essential that roots maintain the capacity to acquire soil water and nutrients. This is reflected by the ability of roots to continue elongation even under severe water deficit conditions albeit at a slower rate [9]. From a physiological perspective, root growth maintenance is predominantly regulated by the plant hormone abscisic acid (ABA). Accumulation of ABA suppresses excessive ethylene production and thereby prevents growth inhibition. ABA is further involved in the processes leading to osmotic adjustment as it promotes the transport of proline to the root apex. At the more basal regions of the root, hexoses are the predominant solutes providing osmotic adjustment and maintaining turgor pressure [reviewed in [10]. From a cellular viewpoint, the processes related to the water deficit response begin with stress perception, followed by signal transduction, and a change in gene expression that finally confers the complex metabolic and physiological alterations necessary to gain stress tolerance [11, 12]. On the molecular level, genes regulated by water deficit can be grouped into two categories. The first group of genes encodes proteins providing direct stress tolerance such as chaperones, transporters, osmolytic and detoxifying proteins, and repair-enzymes [13]. The second category includes proteins involved in stress response by regulating signal transduction and gene expression for instance transcription factors, protein kinases and phosphatases, and other signaling molecules [13]. The high quantity of genes regulated upon water deficit reflects the complexity of the stress response [14]. Nevertheless, details of the translation of environmental changes to metabolic responses i.e. the adjustment of transcriptional and post-transcriptional modifications of metabolic enzymes still remains unclear [12].

In the past, microarray chip hybridization experiments monitored gene expression profiles of maize leaves and roots to elucidate the transcriptional changes upon water deficit [14–19]. Recently developed next-generation sequencing approaches such as RNA sequencing (RNA-Seq) allow fully quantitative gene expression analyses [20] of all 39,656 (FGSv2; [21], release 5b.60) high-confidence maize gene models currently annotated [22]. The digital nature of the method enables the detection of a large dynamic range of expression levels with absolute values and the capture of even subtle gene expression changes [23, 24].

In the present study, a tightly controlled, reproducible experimental setup was applied to expose maize seedlings to water deficit conditions. Young seedlings were grown in paper rolls soaked with polyethylene glycol (PEG) solutions. Previously, it was demonstrated that PEG treatment is an effective way to simulate drought stress conditions occurring in drying soil [14]. Water potentials of -0.2 MPa and -0.8 MPa were used for mild and severe water deficit treatments, respectively. At -0.2 MPa root growth is not or only slightly affected while shoot growth is reduced by half [25]. The lower water potential of -0.8 MPa completely inhibits elongation of maize shoots and leaves while roots continue to elongate consistently [9, 25, 26]. This maintenance of root growth in drying soil is beneficial to plants as they can reach deeper water resources. As top soil layers are prone to drying, it is particularly important for seedlings to adapt to low water potentials [9]. Although maize is most susceptible to drought stress during the flowering period [27], drought conditions during the seedling stage can negatively affect its yield [28].

To gain a better understanding of the early molecular responses to water deficit we utilized an RNA-Seq approach and compared the root transcriptomes of stressed and control maize seedlings. Seedlings were subjected to mild and severe water deficit conditions for 6 h and 24 h. The overall goal of this study was to identify a set of genes involved in initial water deficit responses in maize primary roots. Furthermore, application of mild and severe water deficit conditions at two time points aimed at detecting specifically and commonly regulated genes across treatment intensities and time. This data set will be a resource for future genetic analyses of candidate genes involved in water deficit response in young maize primary roots.

## Results

### Characterization of water deficit treatment and phenotypic responses

**Figure 1:**
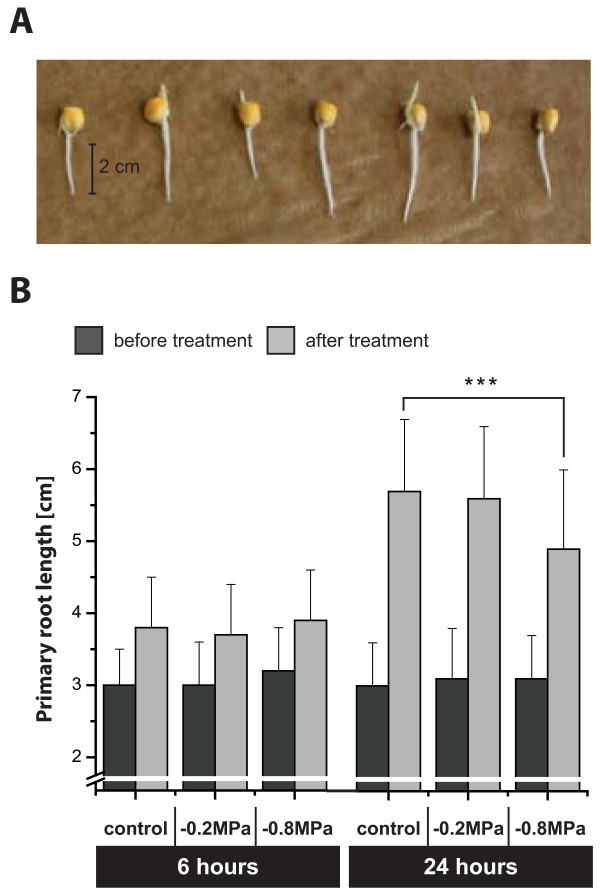
**Root length measurement. A**, Maize (B73) seedlings before treatment. **B**, Primary root length of seedlings before treatment (black bars) and after 6 h and 24 h of control or water deficit treatment (gray bars; water potential of -0.2 MPa: mild deficit, water potential of -0.8 MPa: severe deficit); n = 40, error bars: standard deviation; ***p-value <0.001.

Kernels of the maize inbred line B73 were germinated in paper rolls soaked with distilled water until seedlings had a primary root length of 2 to 4 cm (Figure 1A). For mild and severe water deficit conditions, seedlings were transferred to PEG8000 solution with water potentials of -0.2 MPa and -0.8 MPa, respectively (see Methods). Water deficit treatment was applied for 6 h and 24 h. Each treatment was performed in four biological replicates each consisting of 10 roots.

To analyze phenotypic stress responses, primary root length was measured before and after treatment (Figure 1B). Short-term water deficit did not affect root elongation significantly. On average roots elongated 0.75 cm in 6 h. Similarly, after 24 h of water deficit treatment no differences of root growth between control and mildly stressed seedlings were observed (2.5 cm). However, severe water deficit significantly reduced root elongation (1.8 cm in 24 h) by almost 30% (*p* <0.001).

### RNA sequencing and mapping of maize primary root transcriptomes

To identify genes responsive to water deficit in young maize primary roots, global gene expression was surveyed by Illumina RNA-Seq. On average, the RNA-Seq experiments yielded between 20 and 34 million reads per sample. The raw sequencing data has been deposited at the NCBI sequencing read archive (SRA, [29], Accession: SRP032921). Among all reads, 83 to 90% mapped to unique positions in the maize reference genome (ZmB73_RefGen_v2; Additional file 1). After removal of redundant reads sharing the same start and end coordinate, sequencing direction, and sequence (“stacked reads”), 76 to 78% of the remaining reads mapped uniquely to the “filtered gene set” (FGSv2, release 5b.60; Additional file 1), a set of 39,656 high confidence gene models predicted by a combination of evidence-based and *ab initio* approaches followed by stringent TE filtering [21, 22]. A gene was declared expressed if a minimum of five reads mapped in all four replicates of a sample. As a result, 25,570 genes (64%) of the FGSv2 were expressed in at least one of the experimental conditions. A complete list of expressed genes with normalized expression values is provided in Additional file 2.

### Exploration of differentially expressed genes in response to water deficit

**Figure 2:**
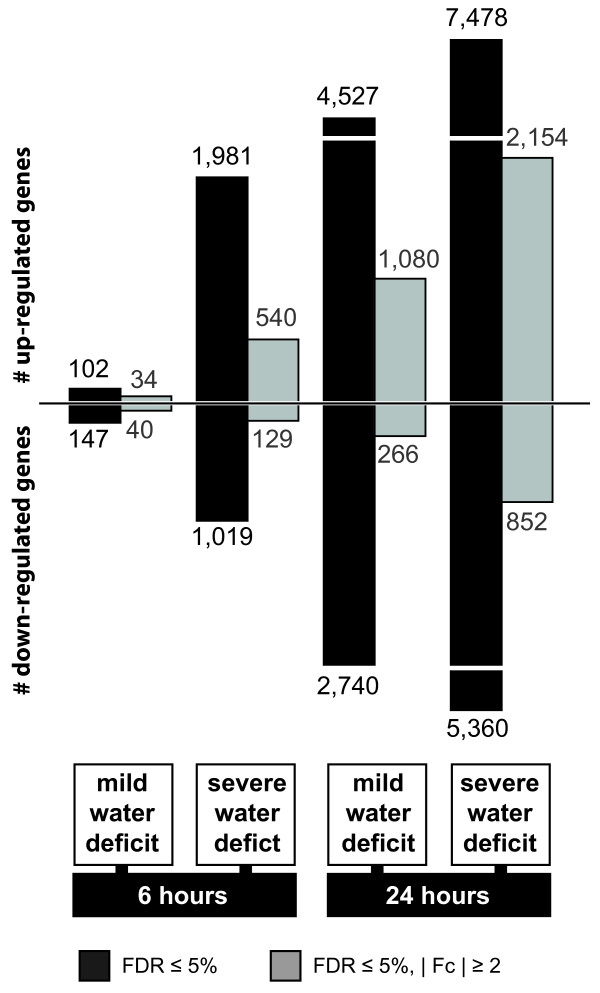
**Number of differentially expressed genes.** Bars represent up and down-regulated genes in the four pairwise comparisons of control groups and water deficit treatments.

To determine genes differentially expressed between control and water deficit conditions four pairwise comparisons of control groups versus the different water deficit treatments (mild deficit, 6 h (1), severe deficit, 6 h (2), mild deficit, 24 h (3), and severe deficit 24 h (4)) were performed. When controlling false discovery rate (FDR) at 5%, 249 and 3,000 genes were differentially expressed in response to 6 h mild and severe water deficit, respectively. After 24 h, the number of differentially expressed genes increased to 7,267 and 12,838 genes for mild and severe water deficit, respectively (Figure 2, black bars; Additional file 2). Small fold-changes (Fc) dominated among differentially expressed genes as about three-quarters of all affected genes display a |Fc| ≤2 (Additional file 3). To specifically focus on genes with a strong response to water deficit only genes with a |Fc| ≥2 were considered in subsequent analyses. This arbitrary cutoff reduced the numbers of differentially expressed genes to 74 (vs. 249) and 669 (vs. 3,000) for 6 h mild and severe water deficit and 1,346 (vs. 7,267) and 3,006 (vs. 12,838) for 24 h mild and severe deficit, respectively (Figure 2, gray bars; Additional file 2).

**Figure 3:**
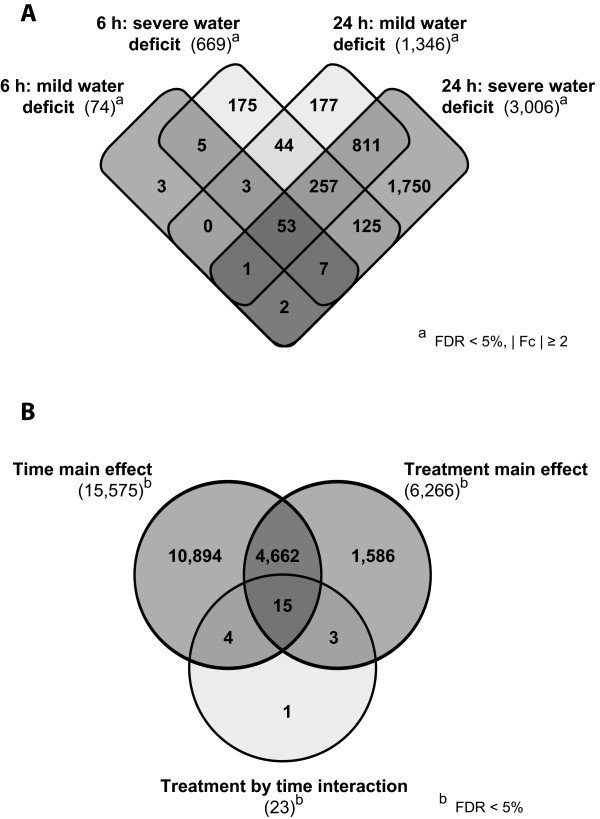
**Overlap between different sets of water deficit responsive genes. A**, Overlap between differentially expressed genes responsive to 6 h mild water deficit, 6 h severe water deficit, 24 h mild water deficit, and 24 h severe water deficit. (The total number of affected genes is given in brackets). **B**, Overlap between differentially expressed genes identified as time main effect, treatment main effect, and treatment by time interaction.

In summary, the results demonstrated that the duration and intensity of water deficit conditions significantly influence the number of responsive genes and the intensity of the response. In line with that observation, the maximal absolute Fc detected increased from 10-fold after short-term mild water deficit to 304-fold after 24 h severe water deficit. However, the average |Fc| was similar under all conditions and ranged between 2.8 and 3.6-fold.The gene expression patterns in response to short-term mild water deficit differed from the patterns in response to the more intense or longer stress treatments. After 6 h of mild stress 46% of differentially expressed genes were up-regulated. In contrast, in the other three comparisons 72 to 82% of the water deficit responsive genes were up-regulated (Figure 2).The overlap between the gene sets of the four comparisons is visualized in Figure 3A. Cross-comparison of responsive genes showed that 83% of genes responding to short-term mild water deficit and 81% of genes responding to short-term severe water deficit are also responsive after 24 h of water deficit. Similarly, 96% and 90% of genes responding to 6 h and 24 h mild water deficit were also responsive to severe water deficit. A set of 53 genes was differentially expressed (|Fc| ≥2) independently of water deficit level and treatment period. Among those, 30 were down and 23 up-regulated in all treatments. Such conservation of regulation direction was observed for most (99%) of the differentially expressed genes overlapping between two or more treatments. All 53 consistently responding genes were included in the set of 74 genes responsive to 6 h mild stress. Only 3 of 74 genes that were differentially regulated after 6 h of mild water deficit were specific for this treatment. After short-term severe water deficit and long-term mild water deficit 175 and 177 genes were uniquely affected, respectively. The highest number of uniquely affected genes was detected for 24 h severe water deficit (1,750, 58% of all responsive genes).

Besides the pairwise comparisons a two-way analysis of variance was performed to determine treatment main effects, time main effects, and treatment by time interactions. This analysis yields genes differentially regulated in response to both water deficit levels, both treatment periods, and to the combination of treatment intensity and duration, respectively. The latter gene set includes genes whose expression does not change initially but changes over time and treatment intensity [30]. As a result of this survey, 6,266 and 15,575 genes displayed treatment main effects and time main effects, respectively. Moreover, 23 genes displayed treatment by time interactions (Additional file 4). Between the three gene sets some overlap was identified: 15 genes were present in all data sets representing 65% (15/23) of the genes that displayed treatment by time interactions. Furthermore, 75% of the genes that showed treatment main effects were among the genes displaying time main effects (Figure 3B).

### Functional categorization of stress responsive genes

**Figure 4:**
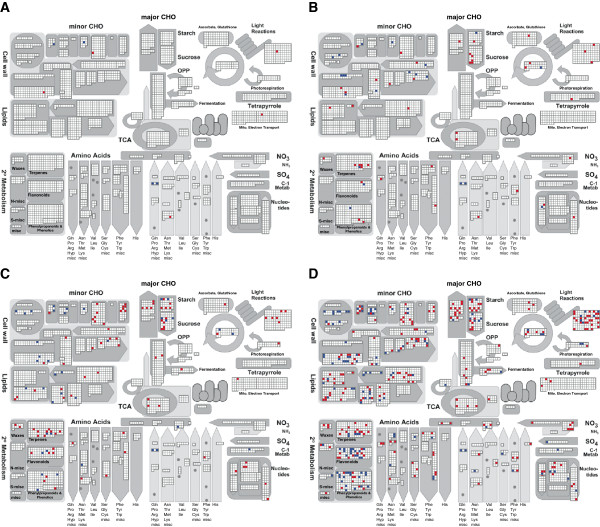
**Overview of metabolic responses to water deficit.** Genes differentially regulated (FDR <5%, |Fc| ≥2) in response to **A**, 6 h mild water deficit, **B**, 6 h severe water deficit, **C**, 24 h mild water deficit, and **D**, 24 h severe water deficit as visualized by Mapman. 74, 669, 1,346, and 3,006 differentially expressed genes corresponded to 80, 715, 1,431, and 3,210 unique transcript identifiers imported in Mapman [31]. Among those, 9, 66, 131, and 380 entities are visible in metabolic overviews. Transcripts which were up and down-regulated are represented in red and blue, respectively.

An overview of the metabolic processes regulated by water deficit was generated with Mapman [31] (Figure 4). Only few of the 6 h mild water deficit responsive genes (FDR <5%, |Fc| ≥2) were included in the metabolic pathways overview, mainly in minor CHO metabolism (sugar and sugar derivate metabolism) and amino acid metabolism (Figure 4A). In response to more intense water deficit treatment, genes involved in major CHO metabolism (biosynthesis and degradation of starch and sucrose), cell wall metabolism and secondary metabolism were differentially regulated (Figure 4B). 24 h of treatment increased the number of responsive genes in all pathway categories with many differentially regulated genes annotated in major and minor CHO metabolisms, cell wall metabolism, lipid metabolism, and secondary metabolism (Figure 4C, D). An additional biochemical pathway analysis with Plant MetGenMAP [32] classified water deficit responsive genes into analogical pathways. In total, responsive genes were involved in 187 different pathways (Additional file 5). Among those, 126 describe biosynthetic processes, primarily biosynthesis of amino acids, carbohydrates, hormones, lipids, and cell wall compounds. The remaining pathways include degradation/assimilation (39; mainly carbohydrates and amino acids), energy generation (17; e.g. TCA cycle, glycolysis), detoxification (1), or a combination of these (10). Of the only four common pathways between the data sets, three describe down-regulation of proline degradation and one up-regulation of methylglyoxal degradation (Additional file 5).

**Figure 5:**
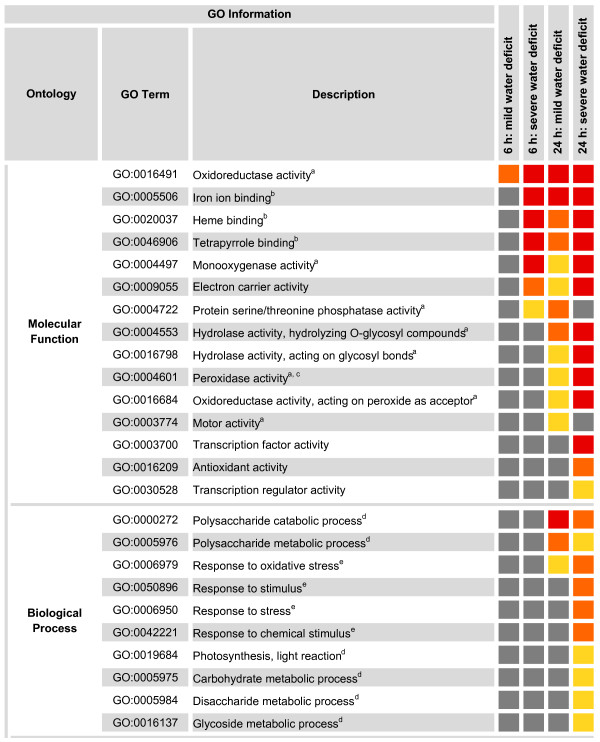
**Cross-comparison of enriched GO terms among differentially expressed genes in response to 6 h mild water deficit, 6 h severe water deficit, 24 h mild water deficit, and 24 h severe water deficit by single enrichment analysis (SEA).** Different colors in the right columns represent the different significance levels of the overrepresentation; yellow: FDR <0.05, orange: FDR <0.01, red: FDR <0.001. Superscript letters indicate higher-ranking GO terms (GO level 2); ^a^Catalytic activity (GO:0003824); ^b^Binding (GO:0005488); ^c^Antioxidant activity (GO:0016209); ^d^Metabolic process (GO:0008152); ^e^Response to stimulus (GO:0050896).

Differentially expressed genes (FDR <5%, |Fc| ≥2) were further functionally classified according to Gene Ontology (GO) terms using agriGO [33]. For 65% (48/74) of the genes responding to short-term mild water deficit and 52% (351/669) of the genes responsive to severe water deficit, at least one GO term was identified. In the 24 h water deficit treatments, GO terms were identified for 46% (622/1,346) of mild deficit and 52% of (1,551/3,000) severe deficit responsive genes (Additional file 2). A singular enrichment analysis (SEA) was performed to discover significantly overrepresented functional categories (FDR <5%) by comparing each annotated gene set to all expressed genes (for 13,618 of the 25,570 expressed genes GO terms were available). The analyses revealed enrichment of terms related to biological processes and molecular function but not of cellular components. In the two 6 h treatment groups one and seven terms were enriched in the molecular function category (Additional file 6). After 24 h of mild and severe water deficit, 15 and 23 GO terms were overrepresented, respectively (Additional file 6). All commonly and uniquely enriched GOs are summarized in Figure 5. The GO term “oxidoreductase activity” (GO:0016491) was overrepresented in all data sets and was the only significant term among 6 h mild deficit responsive genes. Other commonly overrepresented GO terms included the molecular functions “binding” (“iron ion binding” GO:0005506, “heme binding” GO:0020037, “tetrapyrrole binding” GO:0046906), “monooxygenase activity” (GO:0004497), and “electron carrier activity” (GO:0009055). These covered almost all additionally enriched categories of the short-term severe water deficit regulated genes. Most overrepresented GO terms among the genes responding to 24 h mild water deficit were identical to those responding to 24 h severe water deficit including two terms related to hydrolase activity, “peroxidase activity” (GO:0004601), and “oxidoreductase activity” (GO:0016684). After 24 h of severe water deficit treatment 10 additional GO terms were enriched among responsive genes; three categories related to stimulus responses (“response to stimulus” GO:0050896, “response to stress” GO:0006950, “response to chemical stimulus” GO:0042221), four terms referring to C compound metabolism (“carbohydrate metabolic process” GO:0005975, “disaccharide metabolic process” GO:0005984, and “glycoside metabolic process” GO:0016137, “photosynthesis, light reaction” GO:0019684), two categories describing transcriptional regulation (“transcription factor activity” GO:0003700, “transcription regulator activity” GO:0030528), as well as the molecular function “antioxidant activity” (GO:0016209).

GO enrichment analyses were also performed for treatment and time main effects and treatment by time interaction specific genes. For 8,609 (55%) of 15,575 time main effect, 3,826 (58%) of 6,266 treatment main effect genes, and 14 (61%) of 23 treatment by time interaction genes at least one GO term was assigned (Additional file 4). In contrast to the analysis of pairwise comparisons, comparison of these gene sets with the set of all expressed genes by SEA yielded many overrepresented cellular component categories. Among the treatment by time interaction genes no enriched term was found. Among treatment main effect and time main effect genes 10 and 16 categories were overrepresented, respectively (Additional file 7). Each of the terms enriched among treatment main effect genes was included in the enriched time main effect terms. Four terms were connected to protein synthesis (“translation” GO:0006412, “structural constituent of ribosome” GO:0003735, “ribonucleoprotein complex” GO:0030529, and “ribosome” GO:0005840). Further categories were “structural molecule activity” (GO:0005198), the cellular components organelles (“intracellular non-membrane-bounded organelle” GO:0043232, “non-membrane-bounded organelle” GO:0043228), cytosol (“cytoplasmic part” GO:0044444, “cytoplasm” GO:0005737), and “macromolecular complex” (GO:0032991). Furthermore, the time main effect terms included five transport-related terms (“hydrogen ion transmembrane transporter activity” GO:0015078, “monovalent inorganic cation transmembrane transporter activity” GO:0015077, “inorganic cation transmembrane transporter activity” GO:0022890, “proton-transporting ATP synthase complex” GO:0045259, “proton-transporting two-sector ATPase complex GO:0016469) and the nucleosome (GO:0000786) as additional cellular compartment.

### Validation of differentially expressed genes by qRT-PCR

To independently confirm the RNA-Seq results a subset of nine genes differentially regulated in response to all treatments was selected for quantitative real-time PCR (qRT-PCR) analyses (Additional file 8). As a result, 30 of 36 RNA-Seq expression patterns were confirmed i.e. trends of gene expression were identical in the sequencing and qRT-PCR approach. For nine of the 30 data points the analysis did not have enough statistical power to detect significant differences while for 21/30 data points statistically significant differences (Student’s *t*-test: *p*-value <0.05) were measured.

## Discussion

To promote plant survival, roots are often able to continue growth at low water potentials that completely inhibit shoot elongation [9, 25, 26]. As a result, roots might escape dry soil layers and reach deeper water resources. When water availability is so low to be life threatening, yield will be very low even if plants survive. Thus, from an agronomic viewpoint, mere survival of grain crops is irrelevant [34]. Therefore rather moderate water deficits were chosen for the present study. Water potentials of -0.2 MPa and -0.8 MPa were used to simulate mild and severe water deficit conditions, respectively. Short-term (6 h) water deficit treatment did not affect root elongation in the present study. This was expected as marked differences in steady maize root elongation rates between control conditions and low water potentials were observed only after 10 h of treatment [9]. Similarly, 24 h of mild water deficit did not impair primary root growth, while severe water deficit significantly reduced primary root elongation. These findings are comparable to results by Sharp and co-workers [9, 25] whereas Westgate and Boyer [26] measured considerable growth reduction only at water potentials lower than -0.8 MPa. This discrepancy might at least in part be explained by the different maize genotypes that were analyzed in these studies. Since 6 h of water deficit treatment did not affect root growth this experimental period was used to monitor gene expression changes at the initial phase of the water deficit response. Long-term treatment with mild water deficit had likewise no primary root growth effect suggesting that the root is able to tolerate this mild drought. In contrast, severe water deficit decelerated primary root elongation implying that the root had adapted by metabolic alterations.

RNA-Seq technology was utilized to compare the primary root transcriptomes of seedlings treated with low water potentials to those grown under control conditions. The seedlings used for the experiment were selected by their root length rather than by age minimizing variability of gene expression due to developmental differences [35]. In total, 25,570 genes were expressed under at least one of the time/treatment combinations. In the present study, 74 and 669 genes were identified that responded with expression changes to 6 h of mild and severe water deficit, respectively (|Fc| ≥2). After 24 h, 1,981 and 3,006 genes were differentially expressed (|Fc| ≥2) in response to mild and severe water deficit, respectively, reflecting complex acclimatization processes. A similar pattern of an increased number of responsive genes following more intense stress treatment has been reported for maize (Han21) seedling roots by Zheng, et al. [14], who considered 190 uniquely expressed sequence tags, and also for the whole maize (SRG-200) root system [15]. Comparative analyses in this study revealed that most (≥80%) of the short-term water deficit responsive genes were a subset of the long-term water deficit responsive genes. Similarly, a large overlap (≥90%) between mild and severe water deficit responsive genes was detected. An analogous pattern was observed for drought-stressed maize (SRG-200) and barley root systems although stress treatment was more intense in these studies [15, 36]. However, neither maize nor barley leaves showed this pattern [15, 19, 36]. This indicates a root-specific response that is already established after the short period and low intensity of water deficit used in this study. Besides, there is further indication that the drought stress response is organ-specific. A microarray analysis of maize root, leaf, and shoot gene expression changes in response to drought stress revealed only a rather limited overlap between stress regulated genes [15]. In line with that observation the functional categories affected by the transcriptional changes were very distinct across tissues [15]. Similarly, a comparison of differentially expressed genes identified in the present study to genes identified by Kakumanu, et al. [37] via Illumina sequencing of RNAs isolated from basal leaf meristem tissues of maize plants grown under drought stress yielded very little overlap both on gene and functional category level. However, plant developmental stages (seedlings vs. adult plants, reproductive stage) and treatment period (24 h vs. 4 d) were quite different.

Among the water deficit responsive genes identified in this study several GO categories were overrepresented in comparison to all expressed genes. Cross-comparison of these categories resulted in one commonly enriched term, “oxidoreductase activity”. Its child term “monooxygenase activity” was also enriched among three of the four gene sets. Several previous studies detected differential expression of oxidoreductase and monooxygenase genes in response to water deficit in maize, barley, and soybean [14, 18, 36, 38–40]. However, the complexity of these studies was limited to a few hundred or thousand entities and only individual genes or proteins of the oxidoreductase category were identified along with other differentially expressed genes. The reactions catalyzed by oxidoreductases can result in scavenging as well as in the generation of reactive oxygen species (ROS). Differential expression, including both up and down-regulation, of ROS scavengers in response to drought stress has also been shown in Arabidopsis [reviewed in [41]. While ROS can cause cellular damage [42], they also play an important role as signaling molecules [reviewed by [43] and are associated with growth and development [reviewed in [44]. For instance, the expression and protein abundance of an oxalate oxidase that produces hydrogen peroxide increased in stressed maize roots [18, 40] and is probably involved in root growth regulation upon water deficit [45]. Other commonly enriched GO terms among water deficit responsive genes in the present study were “heme binding”, its ancestor term “tetrapyrrole binding” and the functionally related category “iron ion binding”. Likewise, genes annotated in the Mapman category “metal handling” were commonly up-regulated in response to water deficit (data not shown). In wheat roots an increase in total iron content during drought stress was detected [46]. Besides, the level of bound iron in soybean roots was substantially higher under water stress due to sequestration by ferritin proteins [10]. The expression of these and other metal-chelating proteins was also up-regulated in stressed maize roots [18]. Sequestration of metal ions is beneficial as it prevents the formation of the highly toxic hydroxyl radicals via the metal-dependent Haber-Weiss reaction or the Fenton reaction [41]. Additional studies are needed to fully elucidate the complex interaction of ROS metabolism and regulation of gene expression upon water deficit.

After 24 h of both mild and severe water deficit treatment, further enrichment of GO terms related to sugar and general carbohydrate metabolic processes (“carbohydrate metabolic process”, its child terms “polysaccharide metabolic process”, “polysaccharide catabolic process”, and “disaccharide metabolic process” as well as “glycoside metabolic process” and its child term “hydrolase activity”) was detected. Accordingly, many of the long-term water deficit responsive genes were annotated in pathways of C compound metabolisms. Accumulation of carbohydrates in response to water deficit has been reported in several species and plant organs [reviewed in [47]. In maize primary roots, soluble carbohydrates together with proline account for osmotic adjustment which has an essential role in maintenance of root elongation at low water potentials [48, 49]. It has been reported that both increases in proline synthesis and decreases in proline oxidation occur in response to low water potentials to increase proline concentrations [49]. Accordingly, three of four biochemical pathways conserved among water deficit responsive genes to all treatments included down-regulated proline oxidases. Besides functioning in osmotic adjustment, C compounds also participate in signaling and transcriptional and post-translational regulatory processes in metabolic and developmental programs [47] and the regulation of their synthesis/degradation is therefore tight and complex in response to environmental stresses.

An analysis of variance yielded differentially expressed genes in treatment main effect, time main effect, and treatment by time interactions. The highest number of genes (15,575) appeared in time main effect including more than half of all expressed genes. This suggests strong differences between root transcriptomes of seedlings differing in age as seedlings after long-term experiments were 18 h (about 20%) older and on average 1.6 cm (about 42%) longer than seedlings after short-term experiments. This is in accordance with the report of differences in protein abundance between two early stages (5 and 9 DAG) of maize root development in the inbred line B73 [50] which was also studied in the present survey. As most of the treatment-specific genes (4,662 of 6,266) are included in time effect genes, the pairwise comparisons between treatments within each time period might be more accurate. The test for treatment by time interaction could identify genes that do not differ across water deficit conditions initially but develop differences across conditions with longer treatment periods [30]. A comparable low number of 23 genes were assigned to this category. Hence, only a minor fraction of all water deficit responsive genes changed their expression from one time point to the other. This is in line with the large overlap observed between mild and severe deficit as well as between short and long-term treatment responsive genes in the pairwise comparisons.

A GO analysis revealed largely the same overrepresented categories among genes that display time main effects and treatment main effects. About one quarter and one half of the enriched GO terms, respectively, are related to protein biosynthesis including the biological process translation and the ribosome as cellular component. This points to a developmental function of these genes specific for time and for treatment effects as ribosomes are generally considered as housekeeping components of the cell and are involved in growth processes. Genes encoding ribosomal proteins are highly expressed in proliferating, elongating, and differentiating cells [51]. However, it was demonstrated for Arabidopsis that expression of these genes changes in response to abiotic stress [52, 53]. Furthermore, a study in maize indicated that modifications of the translational machinery emerge in response to hypoxic stress [54]. In the present study the same genes functionally related to ribosomes were identified for time main effects and treatment main effects. This supports the concept of these genes playing a double role in normal development/growth and in water deficit response.

## Conclusions

In the present study, hundreds of genes were identified that are differentially expressed in response to water deficits in maize seedling primary roots. The number of responsive genes was dependent on water deficit condition and duration and increased with intensification of treatment. The products of the differentially expressed genes are known to be involved in perception and signal transduction or confer adaptation and tolerance to low water potentials. Analyses of associated GO categories and underlying biochemical pathways connect the water deficit response to ROS and carbohydrate metabolisms and signaling, revealing their complex transcriptional regulation. Further functional analyses of these genes will promote our understanding of the molecular mechanisms underlying root adaptation to water deficit and enable targeted breeding strategies. Thus, more tolerant crop varieties, that can be cultivated with stable yield in dry regions as well as on fields with uneven soil moisture, might be generated.

## Methods

### Plant material and water deficit stress treatment

Seeds of the maize inbred line B73 were surface sterilized and germinated in paper rolls as described in Ludwig, et al. [55]. Four to 5 days (16 h light, 28°C; 8 h dark, 21°C) after germination seedlings with a primary root length of 2 to 4 cm were transferred to new paper rolls soaked with polyethylene glycol (PEG8000 M_r_ 7,300-9,000; Roth, Karlsruhe, Germany) solutions with a water potential of -0.2 MPa and -0.8 MPa for mild and severe water deficit, respectively, or distilled water for control experiments. Because manufactured PEG8000 includes a range of molar masses water potentials could not be calculated directly. Osmolyte concentrations (mol*l^-1^) of PEG solutions with defined mass concentrations (g*l^-1^) were measured with an osmometer (OSMOMAT 030-D, Gonotec GmbH, Berlin, Germany). This data was used to estimate water potentials (water potential [MPa] = concentration [mol*l^-1^] * gas constant [8.314 Pa*l*mol^-1^*K^-1^] * temperature [298.15 K]). Paper rolls with seedlings were incubated in aerated PEG solutions or distilled water for 6 h and 24 h (24°C, 24 h included 8 h darkness, 18°C). Before and after treatment, seedlings were photographed and primary root length was measured using WinRHIZO (http://www.regent.qc.ca/). Whole primary roots were harvested, immediately frozen in liquid nitrogen and stored at -80°C until RNA isolation. Experiments were performed in four biological replicates each consisting of 10 pooled roots.

### RNA isolation and sequencing library preparation

Pooled primary roots were ground in liquid nitrogen, and RNA was extracted as previously described [56]. RNA quality was assessed via agarose gel electrophoresis and a Bioanalyzer (Agilent RNA 6000 Nano Chip, Agilent Technologies, Santa Clara CA, USA). Only samples with an RIN [RNA integrity number; [57] ≥9.1 were used for downstream analyses. The cDNA libraries for Illumina sequencing were constructed in accordance with the protocol of the manufacturer (TruSeq RNA Sample Preparation, Illumina, San Diego CA, USA). For sequencing, four libraries were pooled in one lane of a flow cell. Each library per lane was indexed by one of the adapters AR001, AR008, AR010, or AR011 (Additional file 1). The indexed libraries were loaded onto a flow cell according to an incomplete block design (generated with CycDesigN, http://www.vsni.co.uk/software/cycdesign; Additional file 1). Cluster preparation and single read sequencing were performed according to the manufacturer’s instructions (HiSeq 2000, Illumina, San Diego CA, USA).

### Processing and mapping of Illumina sequencing reads

Raw sequencing reads generated by the Illumina HiSeq 2000 system were initially processed and quality trimmed with SHORE (http://1001genomes.org/software/shore.html). Reads with more than 2 or 5 bases having quality scores ≤3 in the first 12 or 25 bases, respectively, were rejected [58]. Bases with quality scores ≤5 at the 3’ end were trimmed until two succeeding bases with higher quality scores. Reads with ≥2 mismatches in adapter sequences were excluded. Only reads ≥40 bp were retained for subsequent analyses. Finally, adapter sequences were removed. Resulting reads had a length of 40 to 100 bp (≥60% of all reads were 100 bp long) and quality scores of 26 or higher at all base positions. Read mapping was performed with CLC Genomics Workbench (http://www.clcbio.com/products/clc-genomics-workbench/). All high quality reads were mapped to the maize B73 reference genome [RefGen_v2; [21, 22] allowing large gaps of up to 50 kb to span introns. At least 75% of each read had to fit with 90% similarity to the reference to be mapped. Stacked reads i.e. redundant reads sharing the same start and end coordinate, sequencing direction, and sequence were merged into one. The remaining reads were projected to the filtered gene set (FGSv2; [21], release 5b.60) of the B73 reference genome derived from the maize genome sequencing project (MGSP) allowing a maximum of two mismatches for reads ≤56 bp. Longer reads had to fit at least with 80% of their length thereby comprising 90% similarity. Only those reads uniquely mapping to the reference data set were subsequently used for analyses.

### Statistical procedures for analyzing differential gene expression

The read counts were normalized to RPKM (reads per kilobase of exon model per million mapped reads) values [59] and log2 transformed to meet the assumptions of linear models. Further statistical analysis followed the empirical Bayes approach of Smyth [60]. The mean-variance trend for log-counts was estimated and a weight assigned to each observation based on its predicted variance. The weights were then used in the linear modeling process to adjust for heteroscedasticity [61]. To borrow strength across genes in the estimation of the residual error variance, the empirical Bayes approach of Smyth [60] implemented in the R-package *limma* was used. A linear mixed model with lane effect was applied accounting for the lanes as incomplete blocks. The lane effect is considered as random effect, thus allowing the recovery of the inter-block information. The experimental setup allowed four comparisons of control groups against different water deficit levels: mild deficit, 6 h (1), severe deficit, 6 h (2), mild deficit, 24 h (3), and severe deficit 24 h (4). After computing these contrasts, resulting *p*-values of each contrast were corrected for multiplicity using the FDR-approach of Benjamini and Yekutieli [62]. Additionally, a two-way analysis of variance (ANOVA) was performed with treatment main effect, time main effect and treatment by time interaction according to the empirical Bayes approach of Smyth [60] implemented in the R-package *limma*. Computed *p*-values were corrected for multiplicity using the positive false discovery rate [pFDR; [63].

### Gene Ontology (GO) and metabolic pathway analyses

GO functional categories were assigned to differentially expressed genes using the web-based agriGO software [33]. Enriched categories were computed using singular enrichment analysis (SEA) by comparing the list of differentially expressed genes to all expressed genes as described in Du, et al. [64]. Multiple comparison correction [62] was performed and FDR controlled at 5%. Through the SEACOMPARE tool analysis results were combined for cross-comparisons. Differentially expressed genes were assigned to metabolic pathways and subsequently visualized using the Mapman software [31, 65] based on the functional annotation file ZmB73_5b_FGS_cds_2011 [65]. Additionally, genes were assigned to biochemical pathways with the web-based Plant MetGenMAP [32, 66] software according to the MaizeCyc database [version 2.1; [67].

### Quantitative real-time PCR (qRT-PCR) analysis

To confirm gene expression levels detected by RNA-Seq, quantitative real-time PCR was performed in a Bio-Rad CFX 384TM Real-Time System (Bio-Rad, Munich, Germany) using gene-specific oligonucleotides (Additional file 9). The cDNA for qRT-PCR analyses was synthesized from 1 μg total RNA with the qScript cDNA SuperMix (Quanta Biosciences, Gaithersburg, MD, USA) using the same RNA samples as for the cDNA library construction. Each PCR reaction contained 4 μl MESA Blue qPCR™ Mastermix Plus for SYBR Assay no ROX (Eurogentec, Cologne, Germany), 1 μl cDNA sample and 100 nM gene-specific oligonucleotide primers to a final volume of 8 μl. The primer efficiency of each oligonucleotide was calculated using the following dilution series: 1, 1/2, 1/4, 1/8, 1/16, 1/32, 1/64, and 1/128. The relative expression levels of the transcripts were calculated with reference to the housekeeping gene *myosin* (Genbank AC: 486090G09.x1). Significant differences in gene expression levels were determined by a two-sided Student’s *t*-test.

## Electronic supplementary material

Additional file 1: **Overview of the sample distribution within the flow cell, biological replication, RNA-Seq output and mapping results.** (XLSX 14 KB)

Additional file 2: **Comprehensive list of the 25,570 expressed genes, normalized expression values, fold-changes,** ***q*** **-values, and GO annotation.** (XLSX 6 MB)

Additional file 3: **Fold-change distribution of differentially expressed genes (FDR <5%) between water deficit treatment and control groups.** (PDF 171 KB)

Additional file 4: **Comprehensive list of genes identified as time main effect, treatment main effect, and treatment by time interaction specific genes.** (XLSX 1 MB)

Additional file 5: **List of pathways containing genes differentially regulated in response to water deficit.** (XLSX 19 KB)

Additional file 6: **Comprehensive results of the singular enrichment analyses (SEA) among differentially expressed genes in pairwise comparisons between water deficit treatment and control groups.** (XLSX 13 KB)

Additional file 7: **Comprehensive results of the singular enrichment analyses (SEA) of differentially expressed genes in time and treatment main effects and in treatment by time interaction.** (XLSX 12 KB)

Additional file 8: **Comparison of RNA-Seq data and qRT-PCR results.** (XLSX 11 KB)

Additional file 9: **List of oligonucleotide primers used for qRT-PCR experiments.** (XLSX 11 KB)
