# Supplementary material for: Transcriptomic complexity in young maize primary roots in response to low water potentials
Source: BMC Genomics. 2014 Aug 29;15(1):741. doi: 10.1186/1471-2164-15-741 (PMC4174653; doi:10.1186/1471-2164-15-741)
Supplement: Supplementary file 3 — Additional file 3: Fold-change distribution of differentially expressed genes (FDR <5%) between water deficit treatment and control groups. (PDF 171 KB) [file 12864_2014_6473_MOESM3_ESM.pdf]

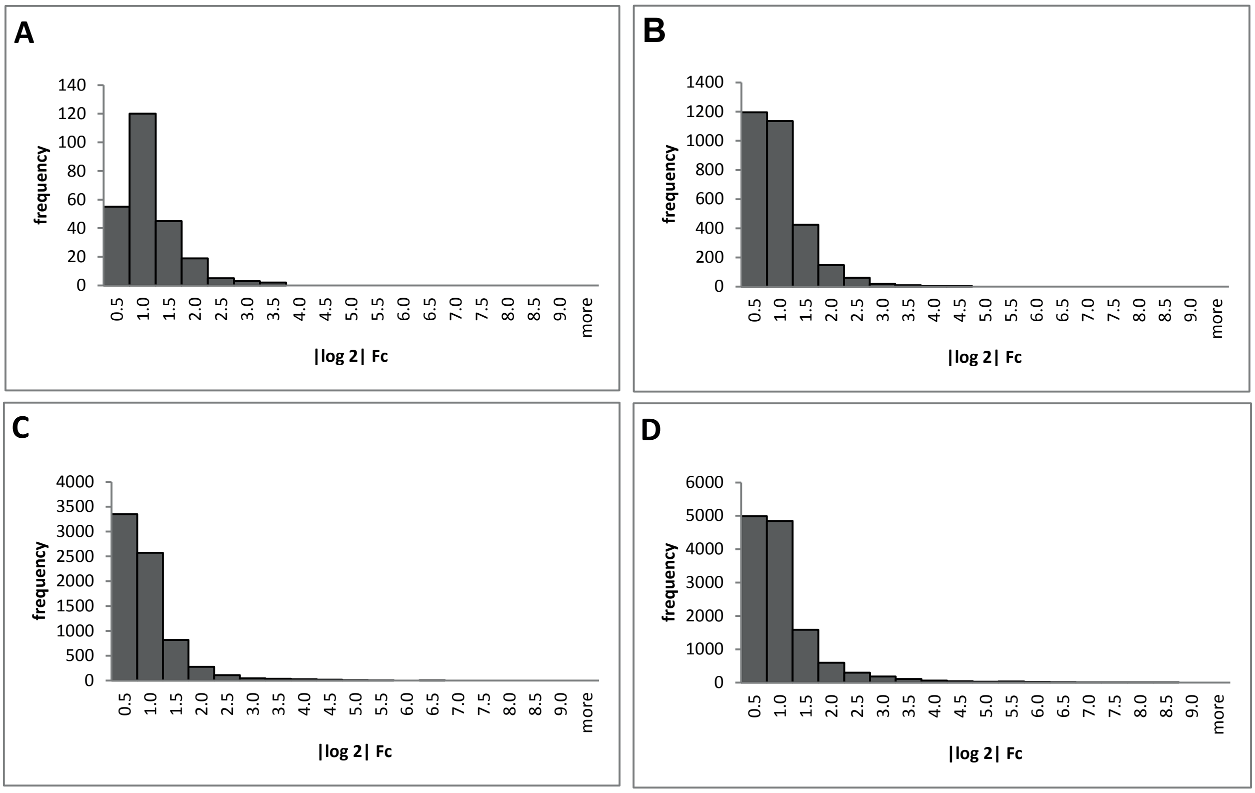

Additional file 3 - Fold change distribution of differentially expressed genes (FDR ≤ 5%) between water deficit treatment and control groups. **A**, 6 h mild deficit, **B**, 6 h severe deficit, **C**, 24 h mild deficit, and **D**, 24 h severe deficit.
